# Our mutual fiends: cancer research in a time of COVID-19

**DOI:** 10.1098/rsfs.2021.0052

**Published:** 2021-10-12

**Authors:** K. Dabin

**Affiliations:** Science Museum, London, UK

**Keywords:** cancer, research, COVID-19, vaccination, cancer research, cancer care

## Abstract

The impact of the COVID-19 pandemic on health services in the UK and around the world cannot be understated. Cancer care services, patient and cancer research communities have been particularly affected. Screening services, treatment and clinical trials have been halted. Research laboratories have been closed or repurposed to tackle the pandemic. Despite these profound setbacks, there are ways in which the pandemic is accelerating areas of cancer research. In the context of a new cancer research exhibition planned by the Science Museum Group, *Cancer Revolution: Science, innovation and hope*, this essay draws out some remarkable parallels between cancer science and the remarkable research effort seeking to tackle the COVID-19 pandemic. Knowledge and therapeutic approaches from seemingly unrelated fields of medical research are opening up new possibilities to control both diseases. As the race to control COVID-19 has shown, the more research angles, disciplines and tools and people we can bring together to tackle the challenge cancer poses, the better our chances of staying ahead of this disease for more of us now and in future.

## 1. A revolution interrupted

The COVID-19 pandemic has had a major impact on the capacity of health systems in the UK, and globally, to continue the delivery of essential healthcare. Unprecedented steps to avoid the rationing of care and an overwhelmed NHS has resulted in millions of existing patients being affected with treatments postponed or cancelled. Furthermore, millions have missed vital opportunities to receive crucial early assessments and diagnoses for health problems. The knock-on effects of such unprecedented disruption to NHS services are likely to be felt within society for years to come^1^.

While colleagues at the Science Museum have been collecting and documenting the pandemic's widespread impacts for posterity, attention has also been turned to curating a new exhibition charting a different but equally remarkable theatre of medical research. *Cancer Revolution: Science, innovation and hope* explores how more of us than ever before are surviving and living longer with cancer, because of advances driven by research^2^. Cancer touches the lives of so many people. The disease causes more than one in four of all deaths in the UK^3^, and the latest research suggests that one in two of us will be diagnosed with cancer in our lifetime, largely a direct result of more of us living longer alongside improvements in how we detect the disease^4^. Prior to the pandemic starting, cancer outcomes had reached a historic milestone: cancer survival has doubled in the last 40 years, and half of the people diagnosed with the disease in England and Wales survive their disease for 10 years or more^5^. The exhibition charts numerous stories of research that have brought about this transformation in cancer care since the start of the twentieth century. From chemotherapy's origins in WW1 mustard gas research to developments in personalized immunotherapies transforming cancer care today, the exhibition weaves together the stories of researchers, clinicians and patients coming together to advance survival. Most of all *Cancer Revolution* seeks to engage our visitors with a paradigm shift not only within cancer science but also in attitudes towards this disease which until very recently was considered largely fatal, to now one which can increasingly be lived with longer and better, managed as a chronic condition. As the pandemic escalated in the early stages of the planning for this exhibition, we learned from anecdotal conversations with researchers and individuals living with cancer (discussions no longer arranged in person but online) and from widespread media reporting, that the pandemic was having an especially devastating impact on the cancer community. It became clear that the exhibition could not fail but take into account the pandemic's impact within its storytelling. The revolution in cancer care is still happening but has been profoundly set back by COVID-19.

Yet, as I explore in this article and within the exhibition, there is hope to be gleaned from this crisis. As devastating as this pandemic has been for individuals faced with cancer, and indeed for the cancer research and charity sector, there are positive if unintended consequences bringing benefits to cancer care and research. At first glance, SARS-CoV-2, a virus, and cancer, a disease of accrued genetic faults causing cells to malfunction, appear wildly unrelated research challenges. Yet, as I discuss, not only are there many parallels between the two diseases but many of the insights gained from decades of research into cancer, alongside those produced at dizzying speed about SARS-CoV-2, that are creating opportunities to advance how we tackle both diseases.

## 2. Cancer research in a time of COVID-19

It cannot be overstated how devastating the pandemic has been for cancer services, the cancer research community, and for many individuals living with the disease. Cancer screening, diagnosis and treatment involves large coordinated teams, many of which were impacted by staffing shortages caused by absences as a direct result of the virus or through redeployment. More than 650 000 people with cancer in the UK experienced disruption to their cancer treatment or care because of COVID-19 [1]. This has been especially problematic for those with advanced cancer whose trials of ‘last hope’ treatments were stopped. At the pandemic's peak, between April and May 2020, more than 1200 trials were delayed worldwide [2]. Our exhibition now shares the experiences of families who have lost loved ones due to the cessation of treatment. Also, following conversations with patients, we will be sharing experiences of the impact of shielding on lives already interrupted by disease. The risk of contracting COVID-19 was found to be particularly high for cancer patients with weakened immune systems, who as a result of treatment or the cancer itself, were less able to fight off infection. Research has shown that cancer patients who contract COVID-19 have a 23% risk of mortality compared to a 6% risk for non-cancer patients^6^.

To showcase the latest research transforming cancer care in the exhibition, we have spoken with scientists from the Crick Institute, Medical Research Council, Cancer Research UK's Manchester, Cambridge and Glasgow Institutes, among many others, and we began to discover first-hand the impact of the pandemic on researchers. Most cancer research laboratories were shut down at the pandemic's peak, and major cancer research centres like the Crick Institute transformed laboratories into testing facilities for local hospitals and care homes, with researchers turning their attention to investigating urgent questions about COVID-19^7^. There have been other unexpected consequences of the pandemic impacting researchers, the reduction in cancer surgery has reduced the pipeline of biopsied tissue used in research, elsewhere there are instances of animal models having to be terminated while investigations were interrupted [3]. A survey of 239 cancer research scientists in the UK in November 2020 found that researchers estimated their work would be set back by an average of six months. The same researchers also estimated that major advances would be delayed for almost 18 months due to numerous effects of the pandemic including laboratory shutdowns, reductions in funding and barriers to enrolling patients on clinical trials^8^. As one scientist reported anecdotally to me, ‘It's been a good chance to catch up on data analysis and papers that need publishing, but much trickier to carry out new research. However, useful zoom calls are, they don't facilitate the in-person, often accidental exchanges of ideas that help you make progress'.

Throughout the pandemic, early detection of the SARS-CoV-2 virus and its variants has been fundamental to managing the pandemic. Early detection in cancer too has long been a critical part of improving patient outcomes, with certain cancers identified at earlier stages having up to three times higher 10-year survival rate than those diagnosed at more advanced stages^9^. The cessation of cancer screening services during the first wave, and reluctance for individuals to trouble their GP for assessments, has resulted in a profound drop in the diagnosis of cancer. Forty-thousand fewer people started cancer treatment in the UK last year^10^. Equally worrying is how hard cancer research and care charities have been hit. Cancer Research UK, our exhibition partner and the UK's largest funder of cancer research, has seen a 30% drop in income (£160 million this year alone) and over the next 3 years, they expect to lose £300 million^11^. Such a reduction inevitably will deal a significant blow to research ambitions with fewer trials or research innovations able to be funded.

## 3. Unintended benefits, parallel universes

As devastating as the pandemic has been, there are positive if unintended consequences with COVID-19 bringing benefits to cancer care and research. From mRNA (messenger ribonucleic acid) vaccine approvals to over-the-phone consultations at a patient's convenience, to innovations in clinical trials, COVID-19 has driven innovation within research and clinical practice. The UK Medicines and Healthcare Products Regulatory Agency (MHRA)'s rapid approval of the BioNTech/Pfizer and Oxford/AstraZeneca COVID-19 vaccines appears to be helping to accelerate approvals of other new drugs, including cancer treatments. Belzutifan, a promising treatment for Von Hippel–Lindau disease that causes renal cell carcinoma, has received a so-called innovation passport from the MHRA, putting it on track to receive an approval decision within 150 days of the final submission of trial data [4].

The traffic is far from one way. Insights gained in decades of research into cancer and its interactions with our immune system are also proving relevant in the fight to control COVID-19. Cancer researchers have played a role in identifying and evaluating potential treatments for overactive immune responses known as ‘cytokine storms’ associated with severe cases of COVID-19. Several drugs being studied as cancer treatments are being evaluated for this purpose, such as acalabrutinib (Calquence), a treatment that blocks the activity of a key protein (BTK) in the immune system^12^. Such crosstalk between seemingly unrelated fields of medical research continues to open-up new possibilities.

While starkly different in biology and impact on the body, there are remarkable parallels between the two diseases. Viruses constantly change, evolving through mutation, creating a challenge our health services are still wrestling with to control variant strains of the SARS-CoV-2 virus. Cancer too is a versatile disease when sufficiently advanced, quick to adapt and become resistant to treatments given to eliminate it. The challenge facing researchers at the heart of both these diseases is evolution, both in understanding what is driving how these diseases change and adapt, and in the quest to innovate new treatments to stay ahead of the ‘moving targets’ that are these diseases. How our understanding of cancer as an evolutionary disease is transforming cancer care is a major theme of *Cancer Revolution*. Cancer's ability to evolve to become metastatic and therapy-resistant is the biggest challenge facing patients, clinicians and researchers, and visitors to the exhibition will encounter the scientists innovative novel approaches to outsmart cancer's ability to adapt. Mirroring the global effort to control the SARS-CoV-2 virus, *Cancer Revolution* shows that it will take multiple approaches to outpace cancer's sophisticated evolutionary mechanisms.

Sequencing evolution in these diseases in action is a key area of science linking cancer and COVID-19. The UK is leading the world in tracking the evolution of the SARS-CoV-2 virus, with the COVID-19 Genomics UK (COG-UK) consortium having already sequenced over 450 000 viral samples at the time of writing^13^. Sequencing has been critical in creating a vast COVID-19 family tree of variants, showing in real time how the virus is evolving at the genetic level. The group's most high-profile discovery came in late 2020, with the detection of the B.1.1.7 strain of SARS-CoV-2, dubbed the Kent strain, found to be 30–50% more contagious because of mutations in the code for the spike protein, which the virus uses to slip inside human cells^14^. *Cancer Revolution* shows how sequencing is having the same remarkable impact on our understanding and treatment of cancer too. In the exhibition, we feature the TRACERx (TRAcking Cancer Evolution through therapy (Rx)) consortium led by Prof. Charles Swanton at The Crick Institute, who over the course of 9 years is tracking how cancer evolves in real time—from diagnosis through to relapse—in 850 patients with the most common form of lung cancer. This is a form of the disease where outcomes urgently need to be improved, as more than 80% of people diagnosed with stage-IV lung cancer do not survive for more than 5 years^15^. Tracking these changes is helping to improve diagnosis, tailor therapies and forestall recurrence, but it requires intensive monitoring beyond what is currently part of clinical practice.

## 4. Viruses and vaccination

There is, of course, a direct relationship between viruses and cancer. As far back as 1911, researchers began to discover that viruses can lead to cancer. In 1964, the Epstein–Barr virus was the first identified to cause cancer in humans [5]. Viral infections are now thought to account for between 12% and 20% of cancers worldwide^16^. Cancer is itself of course not contagious. Viruses, along with other microbes such as *Helicobacter pylori*, a type of bacteria which plays a role in stomach cancers, cause cancer to develop by activating oncogenes (genes that when mutated cause cells to become cancerous) or by inducing chronic inflammation. As with COVID-19, vaccination against cancer-causing viruses is transformational in reducing their impact. One example of this is the human papillomavirus (HPV) vaccine, which accounted for the largest global burden of infection-associated cancers. By over-activating two oncogenes, this commonly transmitted virus is found to decrease the expression of the body's key tumour suppressor protein p53 (known as the ‘Guardian of the Genome’) alongside dysregulating other proteins, all of which eventually might lead to cancer (see endnote 16). In countries with access to HPV preventative vaccination services, there has been a steep decline in mortality caused by cervical cancer (one Scottish study recently showed that the vaccine has reduced cervical disease in women by up to 71%)^17^.

Yet, as we will show in *Cancer Revolution*, viruses are advancing cancer research and treatment in other ingenious ways—and not only with prevention in mind. Viruses' ability to infect cells selectively has in recent years made them strong candidates to be engineered into new cancer-killing therapies. In the exhibition, we feature Prof. Kevin Harrington of the Institute for Cancer Research who has been leading trials of the viral vaccine T-VEC (talimogene laherparepvec) in the treatment of advanced melanoma^18^. T-VEC is a live genetically modified and weakened form of the cold sore herpes virus that is injected directly into tumours to target and kill cancer cells by multiplying inside them. Helpfully, when cells become cancerous they lose some of their viral defences so are particularly susceptible to these tiny infectious agents. The treatment is a two-pronged attack, also sparking the immune system into action to recognize and join in the destruction of the tumour (see endnote 18). Because the virus cannot replicate inside healthy cells, treatments like this have the potential to avoid the severe side effects so often faced by cancer patients. The significant impact of these treatments for patients within trials like Harrington's (patients seeing their tumours shrink before their eyes over days and weeks) has seen T-VEC go on to be an approved NHS treatment for forms of inoperable metastatic melanoma. Further research is exploring how to extend the benefit of this treatment for more patients by combining it with other targeted treatments. Research like this will see more viruses used to treat more patients with a wider range of cancer types than was previously thought possible.

Viruses aside, the innovations in vaccine technology catapulted to the fore to control the pandemic are also opening up other new possibilities for cancer therapies. The Pfizer/BioNTech and Moderna coronavirus vaccines represented a dramatic departure in the field of vaccine research, employing a new technique based on mRNA, the first time this vaccine technology has been approved for use in humans [6]. As well as carrying instructions for making proteins, RNAs help to turn genes on and off, chop up other RNAs, and aid chemical reactions in our cells. COVID-19 mRNA vaccines give instructions to our cells to make a harmless piece of the coronavirus ‘spike protein’. The immune system then recognizes that the protein does not belong there and begins building an immune response against SARS-CoV-2. This same approach—the engineering of mRNA—is also being used to trigger the immune system to target specific cancer cells in a form of a personalized vaccine.

Moderna and BioNTech, among many other biotech companies, are developing tailor-made mRNA cancer vaccines [7]. To produce the vaccine, samples of the patient's tumour and blood are taken and compared using an algorithm. This reveals a series of tumour targets, mutant proteins that are expressed by the cancer, predicted to be useful in training the immune system to attack the disease. The personalized cocktail of immune system awakening and tumour targeting mRNAs are synthesized and given back to the patient, with the hope that they will spark their immune system to better recognize the cancer cells as foreign and will destroy them. Along with personalized vaccines, many of these companies are also trialling ‘off-the shelf’ vaccine candidates targeting more common cancer mutations (see [7]). As the speed of the COVID-19 vaccine roll out has shown, the versatility, speed to manufacture and relatively low cost to produce mRNA vaccines make it an exciting prospect within cancer therapy.

## 5. Hope or hype? Treading a fine line

It is easy to get carried away with the optimism fuelling hopes, and as often hype, surrounding cancer research and that to an extent has also been directed towards the SARS-CoV-2 research effort. Critical histories of scientific and medical research show that there is rarely ever a straightforward narrative of success and progress. COVID-19 vaccines are undeniably a crucial component of the effort to manage the pandemic, yet, focussing on such success alone masks much of the uncertainty and complexity that pervades how research has shaped the pandemic, and omits the role of public health measures among others that have largely been instrumental in managing the pandemic. The unfolding dilemmas over the transmissibility of variants, challenges surrounding the global distribution of vaccines and the problem of vaccine hesitancy, shows how vaccines alone are no ‘magic bullet’.

Likewise, we are acutely aware that *Cancer Revolution's* exhibition narrative runs the same risk. The exhibition narrative treads largely on the side of championing the scientific successes improving cancer care, past and present. ‘Cancer science’ is a hard sell for a family day out at a museum. To instil a sense of hopeful realism among our visitors, and a spirit of agency to take action to become more engaged with cancer science and reduce their own cancer risks (where possible), results in progress taking precedence over the presentation of critical failures, research stagnation and unfulfiled expectations that also reflects much of the history of cancer research [8–10]^19^. Though the exhibition strongly communicates the complexity of cancer as a disease to control, emphasizing that no singular cure or ‘magic bullet’ offers the answer, as with much of the media coverage of COVID-19, the exhibition largely focuses on laboratory-based research approaches rather than the innovations in public health, community-activism and palliative medicine, that cancer care also depends on.

Yet, the exhibition is not entirely without critical reflection. Inequalities within cancer care^20^, brutal impacts of side effects of current cancer therapies, controversies regarding public health campaigns targeting obesity as a cause of cancer, and the increasing cost of therapies for the NHS and self-funding individuals exacerbated by the challenges of developing personalized medicine approaches are all areas touched upon within the exhibition among others. We use interactivity to gently myth-bust much of the exaggerated headlines around causes of cancer the media exposes us to, and we share stories beyond the laboratory bench—such as the work of Dr Philip Crosbie taking lung health screening into Manchester's poorest communities to detect individuals with lung cancer earlier so that they have better outcomes [11]. This screening pilot has now been extended for trials across the UK.

## 6. Conclusion

Through the amazing efforts of NHS staff, charities and other partners, cancer services have robustly returned, with lessons learned from how services were handled during the first wave of the COVID-19 pandemic. Assuming the virus poses no further surprises, *Cancer Revolution: Science, innovation and hope* opens at the Science and Industry Museum in Manchester in October 2021, before launching at London's Science Museum in May 2022. Thanks to COVID-19, the public's interest in medical research has never been higher. The pandemic's wide-reaching effects on cancer care has necessitated the exhibition to evolve, and the team designing it to adapt in multiple ways. Curating an exhibition remotely— away from the objects and opportunities for in-person research visits—has been both a challenge and an opportunity. It has challenged us to be inventive in how we have progressed its design, and in embracing new realities aimed at reducing COVID-19 transmission^21^. We have necessarily updated the exhibition's narrative, so that it now weaves in stories and perspectives about the impact of the pandemic on individuals, health services and in research. The exhibition is still a celebration of how far we have come in tackling cancer, but it now reflects that we cannot take future progress for granted. The COVID-19 pandemic and the exhibition serves as a reminder of the necessity of global, collaborative research and the astounding altruism of patients willing to participate in research—even when they might not experience the benefit of it themselves.

Despite the setbacks the pandemic has posed to the original vision, the exhibition's message of tempered hope for the future outlook of cancer care still rings true. As the race to control COVID-19 has shown, the more research angles, disciplines and tools and people we can bring together to tackle the challenge cancer poses, the better our chances of staying ahead of this disease for more of us now and in future.

## Endnotes

1
https://www.bma.org.uk/media/2841/the-hidden-impact-of-covid_web-pdf.pdf

2 Further information about the exhibition can be found at: https://www.scienceandindustrymuseum.org.uk/whats-on/cancer-revolution

3
https://www.cancerresearchuk.org/health-professional/cancer-statistics/mortality/all-cancers-combined

4
https://scienceblog.cancerresearchuk.org/2015/02/04/why-are-cancer-rates-increasing/

5
https://www.cancerresearchuk.org/health-professional/cancer-statistics/survival#heading-Zero

6
https://news.harvard.edu/gazette/story/2020/10/how-covid-19-impacts-cancer-research-and-treatment/

7
https://www.crick.ac.uk/research/covid-19-response

8
https://www.forbes.com/sites/victoriaforster/2021/01/13/how-has-the-covid-19-pandemic-impacted-cancer-research/?sh=6715498f1dc8

9
https://www.earlydetectioncambridge.org.uk/

10 https://scienceblog.cancerresearchuk.org/2021/02/02/cancer-services-during-covid-19-40000-fewer-people-starting-treatment/#:~:text=But%20the%20sustained%20disruption%20the,across%20the%20UK%20last%20year.

11
https://news.cancerresearchuk.org/2020/07/15/COVID-19-were-having-to-rebuild-and-adapt-because-of-coronavirus/

12
https://www.astrazeneca.com/media-centre/press-releases/2020/calquence-showed-promising-clinical-improvement-in-majority-of-19-hospitalised-covid-19-patients.html

13 Number of viruses sequenced at time of writing 17 April 2021. See https://www.cogconsortium.uk/

14
https://cen.acs.org/analytical-chemistry/sequencing/200000-counting-UK-sequenced-cases/99/web/2021/02

15 The TRACER-x project website can be found here. See http://tracerx.co.uk/

16
https://asm.org/Articles/2019/January/The-Seven-Viruses-that-Cause-Human-Cancers

17
https://publichealthmatters.blog.gov.uk/2018/06/18/ten-years-on-since-the-start-of-the-hpv-vaccine-programme-what-impact-is-it-having/

18
https://www.icr.ac.uk/news-archive/statement-about-nice-decision-on-t-vec-for-treating-inoperable-metastatic-melanoma

19 For example, the failure to reach the goal of ‘curing’ cancer as envisioned by the 1971 War on Cancer declaration, has encouraged researchers, historians and sociologists alike to question the strategy of the ‘war effort’ during the twentieth and twenty-first centuries. See Sonnenschein & Soto [8]. One example of a critical review of cancer research can be found within *A history of lung cancer: the recalcitrant disease* by Timmermann [9]. The author, Carsten Timmerman, asks what happens when medical progress does not seem to make much difference? A further example would be Ilana Löwy's [10] critical account of clinical trials of interleukin-2 at a major French cancer hospital: *Between bench and bedside*.

20 We address structural and personally felt inequalities ranging from the ‘postcode lottery’ of access to cancer services, to the lack of diversity within the work force, to inequalities such as the lack of availability of hair pieces for individuals from non-white communities experiencing hair-loss as a result of cancer treatment, as examples.

21 Examples would be avoiding the use of headsets to deliver audio content and developing enhanced cleaning regimes for tactile objects.

## Data accessibility

This article has no additional data.
